# Sarcopenia and Muscle Dysfunction in Older Adults with Burn Injuries: An Integrated Clinical Perspective

**DOI:** 10.3390/nu18193122

**Published:** 2026-09-23

**Authors:** Joana Santos-Costa, João Páscoa Pinheiro, Luís Cabral, João Santos-Faria, Flávio Ribeiro, Marisa Caetano, Juliana Batista, Henrique Alexandrino, João Paulo Branco

**Affiliations:** 1 Physical and Rehabilitation Medicine Department, Hospitais da Universidade de Coimbra, Unidade Local de Saúde de Coimbra, 3000-075 Coimbra, Portugaljoaobrancofmuc@hotmail.com (J.P.B.); 2 Faculty of Medicine, University of Coimbra, 3004-504 Coimbra, Portugaljlacabral@gmail.com (L.C.); halexandrino123@gmail.com (H.A.); 3 Burn Unit, Hospitais da Universidade de Coimbra, Unidade Local de Saúde de Coimbra, 3000-075 Coimbra, Portugal; 4 Pharmacy Department, Hospitais da Universidade de Coimbra, Unidade Local de Saúde de Coimbra, 3000-075 Coimbra, Portugal; 5 Nutrition and Dietetics Department, Hospitais da Universidade de Coimbra, Unidade Local de Saúde de Coimbra, 3000-075 Coimbra, Portugal; 6 Emergency Department, Hospitais da Universidade de Coimbra, Unidade Local de Saúde de Coimbra, 3000-075 Coimbra, Portugal; 7 Department of Surgery, Hospitais da Universidade de Coimbra, Unidade Local de Saúde de Coimbra, 3000-075 Coimbra, Portugal

**Keywords:** sarcopenia, muscle dysfunction, muscle wasting, older adults, burn injury, hypermetabolism, malnutrition, rehabilitation, nutritional support, functional recovery

## Abstract

Sarcopenia is a progressive skeletal muscle disorder associated with impaired physical function and adverse outcomes in older adults. Burn injury imposes profound hypermetabolic and catabolic stress that may compromise muscle mass, strength, and physical function, particularly in individuals with reduced baseline muscle reserve. This narrative review integrates current evidence on sarcopenia and muscle dysfunction in older adults with burn injuries, addressing baseline vulnerability, burn-related muscle deterioration, diagnostic challenges, clinical implications, and therapeutic strategies. Patients may present with pre-existing sarcopenia or reduced muscle reserve, develop acute sarcopenia during or after burn-related illness, or experience worsening of pre-existing sarcopenia, potentially resulting in an acute-on-chronic trajectory. However, substantial burn-related muscle wasting, weakness, and functional impairment may occur without fulfilling diagnostic criteria for sarcopenia and represent distinct, clinically relevant manifestations of impaired muscle health. Sarcopenia assessment in burn patients is challenging and should be individualized according to clinical context and stage of recovery. Preservation and recovery of muscle health should be addressed throughout burn care through individualized nutritional support, early mobilization, and progressive exercise, with pharmacological strategies playing an adjunctive role. Prospective longitudinal studies are needed to characterize muscle-health trajectories, distinguish sarcopenia from other forms of post-burn muscle dysfunction, and determine their prognostic significance and response to interventions, including effects on functional recovery, independence, and quality of life.

## 1. Introduction

Sarcopenia is conceptualized as a progressive and generalized skeletal muscle disorder with multidimensional diagnostic criteria that integrate loss of muscle strength, impaired muscle quantity/quality and, in its severe form, physical performance [1]. Although there is a well-recognized association with aging (primary sarcopenia), influenced by both environmental and genetic factors, sarcopenia can manifest alongside various chronic and acute conditions (secondary sarcopenia) [1]. Sarcopenia is recognized as a major contributor to functional decline, physical disability, and loss of independence, and individuals with sarcopenia also report significantly poorer health-related quality of life, particularly in domains related to physical function, mobility, activities of daily living, leisure activities, and autonomy [2]. Because untreated sarcopenia correlates with increased mortality rates and significant personal, social, and economic burdens [1,3,4], early identification and intervention are essential.

Burns represent common potentially severe traumatic injuries, with an estimated 11 million people worldwide requiring medical attention each year [5,6]. Advances in critical care, fluid resuscitation, early excision, and infection control have reduced mortality significantly, even for extensive total body surface area (TBSA) burns [5,7,8], as well as hospital admissions and average length of stay [9,10]. However, burn-related morbidity remains substantial [11]; around 30–50% of survivors experience long-term disabilities, including contractures, hypertrophic scars, chronic pain and psychological sequelae [9,12]. Burn severity and prognosis depend on multi-factorial clinical assessment in which advancing age acts as an independent determinant [13,14,15,16]. Notably, burn incidence also rise in older adults, Refs. [11,17] a trend expected to worsen with population ageing, reflecting physiological, cognitive, and sensory declines, compounded by social factors predisposing to domestic injuries [16,18,19,20,21].

Burn injury represents a particularly challenging acute stressor for skeletal muscle. The hypermetabolic and hypercatabolic response to burns is characterized by sustained increases in energy expenditure, proteolysis, inflammation, endocrine and metabolic disturbances, and insulin resistance, while pain, immobilization, surgery, grafting, and nutritional challenges further reduce mechanical and nutritional support for muscle maintenance [12,22]. These effects may be prolonged and are likely to be particularly consequential in older adults [16].

Emerging evidence indicates a bidirectional relationship between burn injury and sarcopenia. Pre-existing sarcopenia is associated with reduced physiological reserve and poorer clinical outcomes, while burn injury induces sustained systemic disturbances that collectively accelerate skeletal muscle wasting [22,23,24,25].

From a clinical perspective, recognizing pre-existing sarcopenia in older burn patients has important implications for risk stratification, multidisciplinary care planning, and resource allocation. Integrating assessments of muscle mass and function could enhance prognostic accuracy and inform tailored interventions aimed at optimizing nutritional support, physical rehabilitation, and overall resilience, ultimately leading to improved long-term results. Many studies in the burn literature report reductions in lean body mass, muscle strength, or physical performance without applying standardized sarcopenia definitions. Furthermore, assessment during the acute phase is complicated by edema, fluid shifts, pain, wounds, grafts, contractures, postoperative restrictions, and critical illness, all of which may affect measurements of muscle quantity, strength, and physical performance.

Consequently, the available literature specifically addressing sarcopenia in older adults with burn injuries appears fragmented and important evidence regarding muscle dysfunction after burn injury does not necessarily establish the clinical impact of formally diagnosed sarcopenia.

The primary objective of this review is to synthesize the available evidence regarding sarcopenia (as part of a larger, clinically relevant continuum of muscle dysfunction) in older adults with burn injuries, with particular emphasis on the interaction between pre-existing vulnerability, burn-related muscle catabolism, diagnosis, functional consequences, and rehabilitation. Secondary objectives are to examine the diagnostic approaches and limitations applicable to this population; characterize the mechanisms contributing to muscle dysfunction; review nutritional, physical, pharmacological, and other therapeutic strategies; and identify priorities for future research.

## 2. Materials and Methods

A narrative literature review was conducted to identify and integrate evidence regarding sarcopenia and muscle dysfunction in older adults with burn injuries.

Electronic databases, including PubMed, Scopus, and Web of Science, were searched for articles published up to March 2026. The search strategy combined terms related to sarcopenia (e.g., “sarcopenia”, “muscle loss”, “muscle strength”, “muscle wasting”, “muscle dysfunction”), burn injury (e.g., “burn”, “thermal injury”), and older adults (e.g., “elderly”, “older adults”, “aging”).

Studies were eligible for inclusion if they: (i) involved adult human participants aged ≥60 years or included older adult subgroups; (ii) addressed sarcopenia, muscle mass, muscle strength, or related functional outcomes; and (iii) examined these outcomes in the context of burn injury, of any depth, cause or extent. Both observational studies (cohort, case–control, and cross-sectional), interventional studies, and relevant review articles were considered. Exclusion criteria included: (i) studies involving exclusively pediatric or non-human populations; (ii) conference abstracts, editorials, perspectives or commentaries without full text; and (iii) studies not addressing muscle-related outcomes or lacking relevance to burn injury.

Titles and abstracts were screened for relevance, followed by full-text assessment where appropriate. Reference lists of relevant articles were also manually screened to identify additional studies and key sources.

A narrative synthesis was undertaken, integrating burn-specific evidence with relevant literature on sarcopenia, muscle wasting, muscle strength, physical performance, nutrition, critical illness, and rehabilitation, as well as consensus statements and guidelines where direct evidence in older adults with burn injuries was unavailable.

Given the narrative nature of this review, no formal systematic selection process or meta-analysis was performed.

## 3. Current Evidence and Clinical Framework

### 3.1. Integrated Framework of Muscle Dysfunction in Older Adults with Burn Injuries

The prevalence of sarcopenia in older adults with burn injuries remains poorly established, partly because the available literature frequently evaluates individual components of muscle health, such as lean body mass, muscle strength, muscle wasting, or physical performance, without applying standardized diagnostic criteria for sarcopenia. Nevertheless, these findings collectively indicate that burn injury occurs in a population with substantial baseline vulnerability and can impose a profound additional insult to skeletal muscle.

We therefore propose an integrated framework in which sarcopenia represents an important diagnostic construct within a broader spectrum of muscle dysfunction. Older adults may enter the burn episode with established sarcopenia, low muscle mass or reduced muscle reserve, frailty, malnutrition, physical inactivity, or multimorbidity. Burn injury and its treatment may then superimpose hypermetabolism, inflammation, proteolysis, immobilization, critical illness, nutritional deficits, surgery, and burn-related physical limitations. The resulting muscle dysfunction may manifest as loss of muscle mass, reduced strength, impaired physical performance, weakness, and functional decline. These manifestations may coexist and interact, but should not automatically be classified as sarcopenia unless accepted diagnostic criteria are fulfilled.

This framework recognizes several possible trajectories. Pre-existing sarcopenia may remain stable or worsen after injury, evolving into an acute-on-chronic state. Patients without established sarcopenia may develop substantial burn-related muscle wasting and weakness without meeting diagnostic criteria, while others may develop acute sarcopenia during or after the acute illness. In critically ill patients, ICU-acquired weakness may further contribute to muscle dysfunction but remains a distinct clinical entity. Similarly, frailty and malnutrition may both contribute to reduced muscle reserve and influence recovery without being synonymous with sarcopenia. The clinical expression of these processes is further modified by burn-specific factors, including pain, edema, wounds, grafts, contractures, postoperative restrictions, and reduced mobility.

#### 3.1.1. Baseline Muscle Vulnerability

Pre-existing sarcopenia may represent an important but frequently unrecognized source of vulnerability in older adults presenting with burn injuries. Opportunistic imaging studies have identified low skeletal muscle mass at hospital admission, while frailty and reduced lean body mass have also been reported in this population [20,26,27,28]. However, because most assessments are performed after injury, findings obtained at or after admission cannot reliably distinguish pre-existing sarcopenia from low muscle reserve or from early changes related to the acute injury, including fluid shifts, immobilization, and catabolic stress. Consequently, the prevalence of pre-existing sarcopenia in older adults with burn injuries remains uncertain.

Aging is associated with progressive changes in skeletal muscle mass and function, including reductions in neuromuscular function, anabolic responsiveness, and regenerative capacity. Sarcopenia is common among community-dwelling older adults, with reported prevalence estimates generally ranging from approximately 10% to 20%, although substantial variability exists according to the population studied and the diagnostic criteria applied [4,29,30,31].

Baseline muscle vulnerability may be further influenced by multimorbidity that commonly coexist in older adults. Diabetes mellitus, obesity, chronic kidney disease, cardiovascular and pulmonary disease, cancer, osteoporosis, and cognitive impairment have each been associated with reduced muscle health or sarcopenia, although the relationships are heterogeneous and may reflect both shared pathophysiological mechanisms and the cumulative effects of chronic disease [1,32]. Many of these conditions are highly prevalent in older adults and are also frequently encountered in burn populations [33,34].

Malnutrition and sarcopenia also frequently coexist and may contribute independently and synergistically to impaired muscle function and reduced physiological reserve. Despite being distinct clinical conditions, both have been associated with poorer physical performance, functional dependence, hospitalization, and mortality in older adults [35,36,37,38,39,40].

Physical inactivity represents another potentially modifiable determinant of muscle health. Reduced mechanical loading contributes to declines in muscle protein synthesis, strength, and physical performance [1,41], and physical inactivity and sedentary behaviour are highly prevalent among older adults [42]. Although pre-injury physical activity levels have been insufficiently characterized in burn populations, pre-existing inactivity may contribute to reduced muscle reserve.

More broadly, impaired muscle strength and physical performance may increase vulnerability to accidental injury by compromising balance, gait, mobility, and the ability to respond rapidly to environmental hazards. Reduced muscle strength and physical performance are established risk factors for falls in older adults, and sarcopenia has been associated with an increased risk of falls and fall-related injuries [43,44]. Although these deficits may plausibly impair the ability to avoid or respond effectively to hazardous situations, direct evidence linking sarcopenia or other forms of muscle dysfunction specifically to burn injury is limited.

#### 3.1.2. Burn-Related Deterioration of Muscle Health

Burn injury imposes a profound systemic stress that can substantially disrupt skeletal muscle homeostasis. Hypermetabolism, inflammation, endocrine and metabolic disturbances, insulin resistance, immobilization, nutritional deficits, and critical illness may act simultaneously to promote muscle protein breakdown, impair anabolic recovery, and contribute to losses in muscle mass, strength, and physical function [45,46,47].

Severe burn injury is characterized by a sustained hypermetabolic and hypercatabolic response driven by persistent sympathetic activation, stress hormone release, and inflammation [45,46]. Resting energy expenditure may increase markedly after major burns, and in severe injury, metabolic alterations can persist beyond the acute phase [45,46]. Sustained elevations in catecholamines and cortisol promote substrate mobilization and protein breakdown, while disturbances in anabolic signalling, including alterations in growth hormone and IGF-1 pathways, may impair protein synthesis and tissue recovery [45,46]. In parallel, pro-inflammatory mediators, including TNF-α, IL-1β, and IL-6, contribute to a catabolic environment and may promote proteolytic pathways and anabolic resistance [47,48]. Burn-associated insulin resistance and hyperglycaemia may further impair metabolic homeostasis and contribute to an adverse environment for skeletal muscle anabolism [45,46]. Together with increased energy and protein requirements, accelerated proteolysis and negative nitrogen balance, these processes contribute to substantial losses of lean tissue and skeletal muscle [45,46].

Immobilization and reduced mechanical loading compound this catabolic response. During acute burn care, mobility may be limited by pain, sedation, critical illness, surgery, graft protection, wound management, edema, weakness, and the development of contractures [49,50,51]. Reduced mechanical loading suppresses muscle protein synthesis, contributes to anabolic resistance, and promotes pathways involved in muscle protein degradation [52,53,54,55]. Older adults may be particularly susceptible to these effects. Experimental bed-rest studies have demonstrated substantial losses of muscle mass and function in older individuals, together with impaired anabolic responses to nutritional stimuli [56,57,58,59]. Thus, immobilization represents not merely a consequence of acute burn care but an additional contributor to deterioration in muscle health.

Critical illness may add a further and partly distinct dimension to this process. Patients with severe burns may require prolonged ICU treatment and may develop ICU-acquired weakness, a generalized weakness arising during critical illness that is not explained by another cause [60,61]. ICU-acquired weakness and burn-related muscle wasting share several contributors, including systemic inflammation, immobility, and metabolic stress, and may coexist with sarcopenia. Nevertheless, these conditions represent distinct clinical entities and should not be used interchangeably.

Sepsis and other complications may further intensify catabolism and interfere with recovery. Treatment-related factors, including prolonged immobilization, corticosteroid exposure, and interventions associated with prolonged critical illness, may also exacerbate muscle dysfunction or limit recovery, particularly in critically ill patients [60,61,62]. These factors are best understood as additional contributors to the overall burden of muscle dysfunction rather than as direct evidence of sarcopenia.

These processes are likely to be particularly consequential in older adults, in whom reduced baseline muscle reserve and age-related impairments in anabolic responsiveness and regenerative capacity may limit recovery from severe catabolic stress [1,63]. Importantly, however, substantial burn-related muscle wasting or weakness does not, in itself, establish a diagnosis of sarcopenia. Patients without established sarcopenia may develop marked deterioration in muscle mass, strength, and function without fulfilling diagnostic criteria, whereas others may develop acute sarcopenia during or following the acute illness. In patients with pre-existing sarcopenia, burn injury may result in further deterioration, potentially producing an acute-on-chronic trajectory. Distinguishing these trajectories remains challenging during acute burn care, when formal assessment of muscle mass, strength, and physical performance may be difficult to perform or interpret.

### 3.2. Diagnostic Assessment of Sarcopenia in Patients with Burn Injuries

Diagnosing sarcopenia in patients with burn injuries requires the application of established diagnostic criteria while accounting for the substantial effects of acute injury and its treatment on muscle mass, strength, and physical performance.

Case finding may be particularly relevant in older adults admitted with burn injuries, as sarcopenia is more common in healthcare settings characterized by acute illness, hospitalization, rehabilitation, and functional decline [64,65]. However, no sarcopenia screening instrument has been specifically validated in patients with burn injuries. Recommendations regarding tools such as the SARC-F questionnaire must therefore be extrapolated cautiously from other populations. The SARC-F assesses strength, walking ability, rising from a chair, climbing stairs, and falls and is recommended by EWGSOP2 as a case-finding tool [1]. Its relatively low sensitivity, however, may result in missed cases when used as a standalone instrument [66], and acute burn-related impairments may substantially affect responses if the questionnaire is administered during hospitalization. Accordingly, a negative SARC-F result should not preclude further diagnostic assessment when clinical suspicion of sarcopenia persists. When feasible, administration with reference to pre-injury function, based on patient or proxy report, may provide exploratory information regarding possible pre-existing functional limitations [67]. Such information may help identify patients in whom pre-existing sarcopenia is plausible but cannot retrospectively establish the diagnosis.

#### 3.2.1. Muscle Strength Assessment

Muscle strength is the primary parameter used to identify probable sarcopenia according to EWGSOP2 [1]. Handgrip strength is the preferred assessment because of its simplicity, reproducibility, and prognostic value, with cut-offs of <27 kg for men and <16 kg for women commonly used to indicate low muscle strength [1]. In patients with burn injuries, however, measurement may be complicated by burns involving the upper extremities, pain, edema, dressings, grafts, contractures, or restricted range of motion [68].

Testing the contralateral, less affected hand may provide a practical alternative when unilateral upper-limb involvement prevents reliable assessment. Results should nevertheless be documented with regard to the hand tested and interpreted in the context of hand dominance, pre-existing asymmetry, bilateral injury, and the stage of recovery. Hand-held dynamometry may also be useful for assessing specific muscle groups, particularly during rehabilitation, although it is not directly interchangeable with handgrip strength for the purpose of applying established diagnostic cut-offs.

When handgrip assessment is not feasible, the chair stand test may be used as an alternative measure of lower-limb muscle strength [1]. However, lower-extremity burns, donor-site morbidity, pain, edema, postoperative restrictions, and reduced mobility may independently impair performance [69]. Thus, low muscle strength during acute burn care may indicate probable sarcopenia, but its interpretation requires careful consideration of whether the measured deficit reflects underlying muscle dysfunction or direct limitations imposed by the injury and its treatment.

#### 3.2.2. Muscle Mass Assessment

Confirmation of sarcopenia requires evidence of reduced muscle quantity or quality [1]. In burn populations, however, assessment is complicated by acute changes in hydration and body composition, as well as practical limitations related to patient positioning, dressings, pain, critical illness, and restricted mobility.

Dual-energy X-ray absorptiometry (DXA) is widely used to quantify appendicular lean mass and is one of the preferred techniques for assessing muscle quantity in sarcopenia research [1]. Its use during acute burn care may nevertheless be limited by the inability to safely transfer or position the patient. In addition, the marked alterations in body water and edema that occur during the acute post-burn period may complicate interpretation of lean soft-tissue estimates, as DXA cannot directly distinguish retained fluid from lean tissue [70,71].

Bioelectrical impedance analysis (BIA) provides a portable and relatively inexpensive alternative and is considered acceptable by EWGSOP2 when DXA is unavailable [1]. However, because BIA-derived estimates are influenced by body-water distribution, the substantial fluid shifts, edema, and changing hydration status associated with burn injury and critical illness may limit their interpretation during the acute phase [71,72,73]. BIA may therefore be more informative once fluid status has stabilized.

Anthropometric measures, including calf circumference and mid-upper arm circumference, may provide pragmatic information when more advanced techniques are unavailable but have limited precision and are not preferred for confirming sarcopenia [1].

Computed tomography (CT) and magnetic resonance imaging (MRI) can provide detailed information on muscle quantity and quality, including muscle cross-sectional area and radiological measures of muscle composition [1,74]. Opportunistic analysis of CT images acquired for other clinical indications enables skeletal muscle assessment without additional imaging and has been applied in older burn patients, in whom CT-derived muscle cross-sectional area and density have been associated with clinical outcomes [27]. However, CT should not be performed solely for sarcopenia assessment because of ionizing radiation and its limited practicality in acutely ill patients. MRI avoids radiation and provides detailed assessment of muscle volume and composition but is generally less feasible in acute or critical illness because of longer examination times and practical constraints related to positioning, monitoring and transport [74].

Ultrasound is a promising bedside technique for assessing muscle quantity and quality. Its portability, absence of radiation, and suitability for patients with limited mobility may be advantageous in burn care. However, insufficient standardization, limited normative data, and the absence of universally accepted diagnostic cut-offs currently preclude its routine use as a primary method for confirming sarcopenia [1,75].

#### 3.2.3. Physical Performance Assessment

According to EWGSOP2, impaired physical performance identifies severe sarcopenia [1]. Recommended assessments include gait speed, the Short Physical Performance Battery (SPPB), the Timed Up and Go (TUG) test, and the 400-m walk test [1]. Among these measures, gait speed is widely used because of its simplicity and prognostic value. However, its application in patients with burn injuries may be limited by lower-extremity involvement, pain, edema, wound dressings, grafting, postoperative restrictions, or contractures and poor performance may therefore reflect not only impaired muscle function but also burn-specific limitations affecting mobility. The 400-m walk test provides information on endurance and physical performance but may be impractical during acute hospitalization. Similarly, the SPPB and TUG may provide useful information regarding lower-extremity function and mobility, but poor performance cannot automatically be attributed to sarcopenia when burn-related factors directly impair standing, walking, balance, or chair transfers.

Repeated chair stand testing also provides information regarding lower-limb function but should be interpreted cautiously when lower-extremity injury or treatment-related restrictions are present. The six-minute walk test is commonly used in burn rehabilitation to assess exercise capacity and functional recovery [76], although it is not part of the EWGSOP2 diagnostic algorithm.

#### 3.2.4. Biomarkers and Emerging Assessment Methods

Current consensus definitions do not incorporate circulating biomarkers into the diagnostic criteria for sarcopenia [1]. In patients with burn injuries, inflammatory and metabolic biomarkers may provide complementary information on mechanisms contributing to muscle dysfunction. Inflammatory mediators, including IL-6 and TNF-α, are increased following severe burn injury and contribute to the systemic inflammatory and catabolic response affecting skeletal muscle [45,47]. Similarly, alterations in endocrine and metabolic pathways, including IGF-1 signaling and insulin resistance, may provide mechanistic information on impaired muscle anabolism following severe burns [45,46]. However, these abnormalities reflect the systemic response to injury rather than sarcopenia specifically and therefore have no established role in its diagnosis.

Emerging methods such as D3-creatine dilution may provide a more direct estimate of skeletal muscle mass than conventional lean-mass measures and have shown associations with MRI-derived muscle mass and clinical outcomes in older adults [77,78]. However, the method remains primarily a research tool and has not been validated in patients with burn injuries or acute illness.

### 3.3. Interventions Targeting Muscle Health in Older Adults with Burn Injuries

No intervention has been specifically established for the prevention or treatment of sarcopenia in older adults with burn injuries. Management should therefore target the modifiable contributors to muscle deterioration throughout the continuum of burn care, while distinguishing evidence derived from sarcopenia populations from that obtained in burn rehabilitation. The strongest rationale supports an integrated approach combining early mobilization and exercise with adequate nutritional support, while pharmacological strategies aimed at attenuating burn-related hypermetabolism and catabolism may have an adjunctive role in selected patients.

#### 3.3.1. Physical Interventions

Physical interventions are central to preserving and restoring muscle health after burn injury. During acute care, early mobilization should begin when clinically feasible and may include active muscle activation, bed mobility, sitting, standing, transfers, and progressive ambulation. Burn-specific clinical practice guidelines support early mobilization and rehabilitation in critically ill adults with burns, although direct evidence that these interventions prevent sarcopenia remains limited [69]. Mobilization should be individualized according to clinical stability, burn location, grafts and donor sites, pain, wound requirements, weight-bearing restrictions, and other factors that may temporarily limit movement.

When voluntary exercise is not feasible, neuromuscular electrical stimulation (NMES) may provide an adjunctive means of muscle activation. Studies in critically ill older and hospitalized adults suggest potential benefits for muscle mass and function, although the evidence remains heterogeneous [79,80]. Its role in patients with burn injuries and specifically in the prevention or treatment of sarcopenia remains uncertain.

Once active exercise is feasible, progressive resistance exercise has the strongest evidence for improving muscle strength and physical function in older adults with sarcopenia [81,82,83]. Aerobic, balance, and functional training may provide complementary benefits, supporting a multicomponent approach adapted to the individual’s baseline function, injuries, clinical trajectory, and rehabilitation goals [82,83].

Exercise training is also well established in burn rehabilitation. Reviews and clinical studies support resistance and combined exercise programmes for improving muscle strength, exercise capacity, and physical function following burn injury [84,85,86,87]. However, these studies have generally not applied established diagnostic criteria for sarcopenia and have rarely focused specifically on older adults. Thus, the available evidence supports exercise for post-burn muscle and functional recovery but does not establish its efficacy for the prevention or treatment of sarcopenia specifically in older burn patients.

#### 3.3.2. Nutritional Support

During the acute phase of major burn injury, energy and protein requirements increase. Energy requirements should preferably be individualized using indirect calorimetry when feasible, particularly in severe burns, while avoiding both underfeeding and overfeeding [88,89]. When indirect calorimetry is unavailable, predictive equations may be used cautiously as their accuracy may be limited by the dynamic metabolic response to burn injury [88,89]. Early enteral nutrition is recommended in patients with major burns when oral intake is insufficient or not feasible [88,89,90]. Nutritional support should be introduced and advanced with appropriate clinical and metabolic monitoring, particularly in frail or malnourished older patients, considering the risk of refeeding syndrome and the presence of organ dysfunction, fluid imbalance, or impaired glucose tolerance [88,89,90].

Adequate protein provision is particularly important for limiting negative nitrogen balance and supporting tissue repair. Protein requirements in adults with major burns are substantially higher than those of healthy older adults; however, targets derived predominantly from general burn populations should not be extrapolated uncritically to frail older patients and should be individualized according to burn severity, nutritional status, organ function, tolerance, and clinical trajectory [88,89,90]. In older adults, adequate high-quality protein intake may additionally help address age-related anabolic resistance, although evidence for specific protein or amino-acid supplementation strategies in older burn patients is lacking.

Micronutrient deficiencies should be identified and corrected, and supplementation may be required in patients with major burns because of increased losses and metabolic demands [88,89]. However, nutritional strategies shown to support burn recovery or general sarcopenia management should not be assumed to specifically prevent or reverse sarcopenia in older burn patients in the absence of direct evidence.

Nutrition and exercise should be considered complementary components of muscle-health management: nutritional support provides substrates for tissue maintenance and repair, whereas progressive muscle loading provides an anabolic stimulus. However, optimal combined strategies for preventing or treating sarcopenia specifically in older adults with burn injuries have not been established.

#### 3.3.3. Pharmacological Interventions

Pharmacological strategies may complement exercise and nutritional interventions by attenuating the hypermetabolic and catabolic response to severe burn injury [91]. However, no pharmacological treatment is currently established specifically for sarcopenia in older burn patients, and improvements in metabolic parameters or lean body mass should not be interpreted as treatment of sarcopenia unless accompanied by appropriate assessment of muscle strength and function [1,92].

Oxandrolone, a synthetic anabolic androgenic steroid, has been used after severe burns to counteract protein catabolism and promote recovery of lean tissue. Available evidence suggests potential benefits on nitrogen balance and lean body mass, although clinical practice remains heterogeneous and evidence specifically addressing older adults is lacking [92,93,94]. Its use should therefore be individualized, considering hepatic and androgenic adverse effects, contraindications, and regulatory context.

Propranolol targets the sustained catecholamine-mediated response to severe burns. Beta-adrenergic blockade can reduce tachycardia, resting energy expenditure, lipolysis, and other components of post-burn hypermetabolism, with potential effects on protein catabolism [92,94,95]. However, evidence that these metabolic effects translate into prevention or treatment of sarcopenia is lacking, particularly in older adults, in whom cardiovascular tolerance and polypharmacy require careful consideration.

Insulin may improve glycemic control and influence protein metabolism in the hypermetabolic burn state, but its role should remain primarily the management of burn-associated metabolic disturbances rather than sarcopenia [92,96].

Overall, pharmacological interventions should be considered adjuncts targeting specific components of burn-induced hypermetabolism and catabolism, rather than substitutes for nutritional support, mobilization, and progressive exercise. Whether they prevent acute sarcopenia or improve clinically relevant muscle outcomes in older burn patients remains uncertain.

#### 3.3.4. Emerging Therapeutic Approaches

Several emerging therapies target pathways involved in muscle anabolism, inflammation, mitochondrial dysfunction, and regeneration, but their potential role in older adults with burn injuries remains largely unexplored. Among muscle-directed agents, bimagrumab, an activin type II receptor pathway inhibitor, has been investigated in older adults with sarcopenia. Although treatment increased lean body mass, improvements in physical performance were not consistently demonstrated, highlighting that gains in muscle mass do not necessarily translate into functional recovery [97]. Bimagrumab has not been evaluated for burn-related muscle wasting.

Other potential therapeutic targets derive primarily from mechanistic and preclinical evidence. Alterations in skeletal muscle mitochondrial function, inflammation, and regenerative pathways provide a biological rationale for interventions targeting these mechanisms [98,99] but evidence remains insufficient for clinical application.

### 3.4. Clinical and Functional Implications

Loss of muscle mass, strength, and physical function may have important consequences for recovery after burn injury, particularly in older adults. Sarcopenia is associated with adverse outcomes across older, surgical, and critically ill populations, including functional decline, prolonged hospitalization, loss of independence, and mortality [1,99]. However, evidence specifically examining sarcopenia defined according to accepted diagnostic criteria in older burn patients remains scarce and outcomes associated with post-burn muscle wasting or low muscle mass should not automatically be attributed to sarcopenia.

Available burn studies nevertheless suggest that reduced muscle reserve is clinically relevant. Low skeletal muscle mass identified on admission imaging has been associated with adverse outcomes, including longer hospitalization, in older burn patients [27]. More broadly, severe burn injury can result in prolonged loss of lean tissue, weakness, and impaired physical function, while advanced age and frailty are independently associated with poorer burn outcomes [12,15]. These observations support the clinical importance of muscle health but do not establish an independent effect of sarcopenia on burn-related morbidity or mortality.

Functional consequences may be particularly important in older survivors. Persistent weakness and reduced physical performance may limit mobility, activities of daily living, and participation, potentially affecting the ability to return to pre-injury levels of independence. These effects coexist with burn-specific determinants of function, including pain, scarring, contractures, graft and donor-site morbidity, and psychological consequences. Longitudinal assessment is therefore important to determine whether persistent functional impairment reflects ongoing muscle dysfunction, established sarcopenia, burn-specific limitations, or a combination of these factors.

Patient-reported outcomes may complement objective muscle and performance measures by capturing the impact of these changes on participation and health-related quality of life. Although the Sarcopenia Quality of Life questionnaire (SarQoL^®^) has been developed for individuals with sarcopenia, it has not been validated specifically in burn populations and should not replace burn-specific or generic patient-reported outcome measures.

## 4. Discussion

This review highlights an important distinction between sarcopenia and the broader spectrum of muscle dysfunction that may occur in older adults with burn injuries. Although burn-related muscle wasting, weakness, and functional impairment are well recognized, the prevalence and clinical significance of sarcopenia defined according to established diagnostic criteria remain poorly characterized in this population. Much of the available burn literature evaluates isolated measures of muscle mass, lean body mass, strength, or physical performance rather than the multidimensional construct required for the diagnosis of sarcopenia. Accordingly, these findings provide substantial evidence that muscle health deteriorates after burn injury, but considerably less evidence regarding the incidence, natural history, or prognostic significance of sarcopenia itself.

The integrated framework proposed in this review places these findings within a longitudinal clinical trajectory. Older adults may enter the burn episode with established sarcopenia or with varying degrees of baseline muscle vulnerability related to ageing, multimorbidity, malnutrition, physical inactivity, or frailty. Burn injury then superimposes a major catabolic stress characterized by hypermetabolism, inflammation, proteolysis, metabolic disturbances, immobilization, nutritional challenges, and, in some patients, critical illness. The resulting deterioration may therefore follow several trajectories: patients without pre-existing sarcopenia may develop substantial muscle wasting and weakness without fulfilling diagnostic criteria; some may develop acute sarcopenia; and patients with established sarcopenia may experience further deterioration consistent with an acute-on-chronic trajectory. Frailty, malnutrition, and ICU-acquired weakness may coexist with and influence these trajectories but should remain conceptually distinct from sarcopenia.

This distinction also has implications for diagnosis. Application of conventional sarcopenia algorithms during acute burn care is complicated because each diagnostic domain may be influenced by the injury itself. Strength testing may be limited by pain, edema, upper- or lower-extremity injury, grafts, donor sites, and movement restrictions; estimates of muscle quantity may be affected by fluid resuscitation and rapidly changing hydration; and physical performance may be constrained by wounds, contractures, postoperative precautions, weight-bearing restrictions, or critical illness. Thus, an abnormal measurement obtained during hospitalization cannot necessarily be interpreted in the same way as an equivalent measurement in a stable community-dwelling older adult. Similarly, retrospective information on pre-injury function may help identify baseline vulnerability but cannot establish a previous diagnosis of sarcopenia.

Rather than identifying a single preferred assessment method for all patients, the available evidence supports an individualized and longitudinal approach. The modality selected should reflect the anatomical distribution of the burn, clinical stability, fluid status, mobility restrictions, and feasibility of repeated measurements. Where acute injury prevents reliable assessment, reassessment as recovery progresses may be particularly informative. Serial measurements of strength, muscle quantity or quality, and physical performance could help characterize the direction and magnitude of change and, potentially, distinguish transient burn-related impairment from persistent muscle dysfunction or evolving sarcopenia. However, the optimal timing, combination of measures, and clinically meaningful thresholds for change have not been established in burn populations.

The same caution is required when interpreting therapeutic evidence. Early mobilization, progressive resistance exercise, and adequate nutritional support have strong physiological and clinical rationales for preserving muscle health, but their efficacy specifically for preventing or treating sarcopenia in older burn patients has not been demonstrated. Burn rehabilitation studies support exercise for improving strength and physical function, whereas sarcopenia literature supports resistance and multicomponent exercise in older adults; these complementary bodies of evidence justify an integrated approach but should not be considered direct evidence of efficacy for burn-associated sarcopenia. Similarly, adequate energy and protein provision are essential components of major burn care and are particularly relevant in older adults with anabolic resistance, yet optimal nutritional prescriptions specifically targeting sarcopenia in this population remain unknown.

Pharmacological interventions require an even clearer distinction between modification of burn hypermetabolism and treatment of sarcopenia. Agents such as oxandrolone, propranolol, and insulin may influence specific metabolic or catabolic pathways after severe burns, but evidence that these effects translate into improvements across the diagnostic domains of sarcopenia is lacking, particularly in older adults. Emerging muscle-directed therapies are similarly insufficiently supported for application in burn-associated muscle dysfunction.

The clinical relevance of this framework extends beyond diagnostic classification. Low muscle reserve, weakness, and impaired physical performance are likely to influence rehabilitation and functional recovery even when formal criteria for sarcopenia are not fulfilled. Nevertheless, current evidence does not establish whether sarcopenia independently predicts mortality, complications, discharge destination, long-term independence, or health-related quality of life in older burn survivors.

Several limitations of the current evidence should be acknowledged. First, direct studies of sarcopenia in older burn populations are scarce, requiring this narrative review to integrate burn-specific studies with evidence from geriatric, critical care, nutritional, and sarcopenia populations. This approach is useful for constructing a clinically coherent framework but necessarily limits the certainty with which findings from other populations can be extrapolated to older burn patients. Second, heterogeneity in burn severity, age thresholds, timing of assessment, measurement techniques, and terminology limits comparison between studies. Most importantly, studies frequently use low muscle mass or individual functional measures without applying consensus diagnostic criteria for sarcopenia. Finally, as a narrative review, the present study did not employ a formal systematic selection process or meta-analysis, and the conclusions should therefore be interpreted as an integrated synthesis of the available evidence rather than quantitative estimates of effect.

Future research should prioritize prospective longitudinal studies and apply standardized sarcopenia definitions, document pre-injury functional status where possible, and assess muscle strength, quantity or quality, and physical performance at clinically meaningful time points. Particular attention should be given to distinguishing pre-existing, acute, and acute-on-chronic sarcopenia from isolated low muscle mass, burn-related muscle wasting, frailty, malnutrition, and ICU-acquired weakness. Core outcomes should include not only mortality and length of stay but also complications, discharge destination, mobility, activities of daily living, participation, return to previous living arrangements, and health-related quality of life. Ultimately, intervention studies are needed to determine whether early identification of deteriorating muscle health and integrated nutritional and rehabilitation strategies can modify these trajectories and improve outcomes in older burn survivors.

## 5. Conclusions

Older adults with burn injuries are particularly vulnerable to deterioration in muscle health because age-related reductions in muscle reserve and regenerative capacity converge with the profound hypermetabolic, inflammatory, nutritional, and immobilization-related effects of burn injury. This interaction may result in substantial losses of muscle mass, strength, and physical function, with important implications for rehabilitation, independence, and long-term recovery.

Muscle health should therefore be considered throughout the continuum of burn care, from baseline vulnerability through acute hospitalization and subsequent rehabilitation. Early identification of reduced muscle reserve, repeated assessment of muscle mass and function when clinically feasible, individualized nutritional support, early mobilization, and progressive exercise provide a pragmatic framework for preserving muscle health and promoting functional recovery. Pharmacological strategies may complement this approach in selected patients by targeting burn-related hypermetabolism and catabolism, although evidence specific to older adults remains limited.

Within this broader framework, sarcopenia represents an important but distinct clinical trajectory and should be diagnosed using accepted criteria rather than inferred from muscle wasting or weakness alone. Future longitudinal studies should determine how different trajectories of muscle health evolve after burn injury and whether integrated, targeted interventions can preserve muscle function, enhance recovery, and improve independence and quality of life in older burn survivors.

## Data Availability

No new data were created or analyzed in this study. Data sharing is not applicable to this article.

## Abbreviations

The following abbreviations are used in this manuscript:

BIA	Bioelectrical Impedance Analysis
CT	Computed Tomography
DXA	Dual-Energy X-ray Absorptiometry
EWGSOP2	European Working Group on Sarcopenia in Older People 2
ICU	Intensive Care Unit
IGF-1	Insulin-like Growth Factor 1
IL	Interleukin
MRI	Magnetic Resonance Imaging
NMES	Neuromuscular Electrical Stimulation
SARC-F	Strength, Assistance with walking, Rise from a chair, Climb stairs, and Falls questionnaire
SarQoL	Sarcopenia Quality of Life Questionnaire
SPPB	Short Physical Performance Battery
TBSA	Total Body Surface Area
TNF-α	Tumor Necrosis Factor alpha
TUG	Timed Up and Go (test)
